# Impact of diabesity phenotype on cardiovascular diseases, major cardiovascular events and all-cause mortality

**DOI:** 10.1038/s41598-023-38221-7

**Published:** 2023-07-12

**Authors:** Kamran Mehrabani-Zeinabad, Fahimeh Haghighatdoost, Noushin Mohammadifard, Jamshid Najafian, Masoumeh Sadeghi, Maryam Boshtam, Hamidreza Roohafza, Fatemeh Nouri, Dagfinn Aune, Nizal Sarrafzadegan

**Affiliations:** 1 Isfahan Cardiovascular Research Center, Cardiovascular Research Institute, Isfahan University of Medical Sciences, Isfahan, Iran; 2 Interventional Cardiology Research Center, Cardiovascular Research Institute, Isfahan University of Medical Sciences, 81745-15, Isfahan, Iran; 3 Pediatric Cardiovascular Research Center, Cardiovascular Research Institute, Isfahan University of Medical Sciences, Isfahan, Iran; 4 Hypertension Research Center, Cardiovascular Research Institute, Isfahan University of Medical Sciences, Isfahan, Iran; 5 Cardiac Rehabilitation Research Center, Cardiovascular Research Institute, Isfahan University of Medical Sciences, Isfahan, Iran; 6 Heart Failure Research Center, Cardiovascular Research Institute, Isfahan University of Medical Sciences, Isfahan, Iran; 7 Department of Epidemiology and Biostatistics, School of Public Health, Imperial College London, London, UK; 8 Department of Nutrition, Oslo New University College, Oslo, Norway; 9 Faculty of Medicine, School of Population and Public Health, University of British Columbia, Vancouver, Canada

**Keywords:** Cardiology, Risk factors, Disease prevention

## Abstract

To investigate the longitudinal association of different phenotypes of diabetes and obesity with the incidence of cardiovascular disease (CVD), CVD- and all-cause mortality. A total of 5432 adults, aged ≥ 35 years and free of CVD were included in this cohort study. Diabesity phenotypes were defined in six categories based on the presence of diabetes (normal (NG), prediabetes and diabetes) and obesity (obese, non-obese). Fasting blood sugar, 2-h post prandial glucose, or using anti-diabetic medicines were used to define diabetes, and body mass index and waist circumference were used to define obesity. Cox proportional hazards models were used to estimate hazard ratios (HRs) for incident CVD, CVD- and all-cause mortality across these categories. After a median follow-up of 11.25 years, 819 CVD cases, 181 CVD deaths and 488 all-cause deaths occurred. In multivariable-adjusted models and irrespective of obesity definition, the phenotypes of normal glucose-obese, prediabetes-obese and pre-diabetes-non obese were not associated with CVD incidence in comparison with NG-non obese phenotype, however, the phenotypes of diabesity, either defined by general or abdominal obesity, were associated with increased risk of incident CVD events (HR = 1.42, 95% CI 1.01, 1.99, and HR = 1.46, 95% CI 1.07, 1.98, respectively). These findings were sex-specific and only in men with a phenotype of abdominal obesity-diabetes, a positive link was observed for CVD incidence (HR = 1.60, 95% CI 1.01, 2.52). No significant association was found between diabesity and death from CVD or all causes. Diabesity is a predictor of CVD and stroke incidence, but not CVD or all-cause mortality, among Iranians. This association is more pronounced amongst men than women.

## Introduction

Diabetes mellitus (DM) is a major health problem worldwide and its prevalence is projected to reach 783 million by 2045, up from 537 million in 2021^1^. According to the IDF report, DM caused 1 death every 5 s in 2021 and over 75% of adults with diabetes live in low- and middle-income countries^2^. In a nationally representative cross-sectional survey of Iranian adults aged 35–70 y, almost 15% and 25% of the population respectively had DM and pre-diabetes between 2014 and 2020^3^. In parallel with an increase in the prevalence of DM, the global prevalence of obesity has roughly tripled since 1975^4^. Similarly, the prevalence of overweight/obesity in Iran rose by 40% over 5 years (from 22% in 2011^5^ to 59% in 2016^6^).

Both DM and insulin resistance are caused by excess fat mass and contribute to the development of cardiovascular diseases (CVD)^7^. An earlier onset of DM is associated with greater morbidity than later onset DM. In addition, patients with DM have a poorer prognosis and higher mortality compared to individuals without DM^8^. There is also strong evidence linking obesity, particularly abdominal obesity, to the onset of CVD risk factors, CVD events and mortality^9–12^. The majority of DM patients are obese at the time of DM diagnosis^13^. Owing to common risk factors, obesity and DM are closely interconnected and the term “diabesity” was coined, which proposes a causal pathophysiological relationship between two conditions. Diabesity may disrupt cardiac function through cardiac, metabolic, inflammatory and neurohumoral modifications^14^. In addition, elevated blood glucose, but under the threshold of DM, may also increase CVD risk^15^. On the other hand, the coexistence of obesity and DM or even elevated blood glucose may synergistically disrupt cardiovascular function.

So far, limited evidence on the association between diabesity and CVD and mortality is available. In a recent prospective cohort study of Chinese, in comparison with subjects with normal glucose test tolerance and normal body weight, those with diabesity had the greatest risk for the development of CVD, whereas no association was found in prediabetic obese individuals^16^. These findings were independent of DM definition, and when participants were categorized based on abdominal obesity, similar associations were observed^16^.

The association of diabesity with CVD and mortality risk needs to be studied in different populations. Indeed, ethnicity, the pattern of obesity, lifestyle factors, the socioeconomic status of the population and access to health care services may result in heterogeneous findings and consequently differences in public health recommendations. However, there is a paucity of research in this regard. Since patients with DM are frequently obese, exploring the risk of diabesity-related CVD may improve our knowledge with respect to risk stratification and the management of cardiovascular events. Therefore, in the present study, we investigated the longitudinal association of diabesity with the incidence of CVD, CVD and all-cause mortality in a large population of Iranians. We also explored whether the associations differed by the definition of obesity (general vs. abdominal obesity).

## Methods

### Study design

This study is a secondary analysis of the Isfahan cohort study (ICS). The ICS is an ongoing longitudinal population-based prospective cohort study. The ICS was conducted in three central cities of Iran (Isfahan, Najaf-Abad and Arak), and recruitment started in 2001 and participants were followed up by Isfahan Cardiovascular Research Center (ICRC), a WHO-collaborating center^17^ with the primary aim of reducing CVD prevalence. Participants were randomly selected using a two-stage cluster sampling method. The inclusion criteria were: being Iranian, aged 35 or older, mentally competent, and not pregnant. The exclusion criteria were: having CVD events at baseline. All participants were interviewed by trained health professionals and data regarding age, gender, education level, marital status, smoking status, physical activity, anthropometric measures and biochemical markers were collected. Every six years, all of the participants were invited for full medical examination and blood sampling for further evaluations. Detailed information about ICS has been provided elsewhere^17^.

### Anthropometric measurements

Height was measured using a nonelastic meter while the subject was barefoot and standing in a normal position and recorded to the nearest 0.5 cm. Weight was measured on a scale while subjects were in light clothing and recorded to the nearest 100 g. Waist circumference (WC) was measured at a level midway between the lower rib margin and the iliac crest using a tape horizontally fixed around the body using a nonelastic meter. Body mass index (BMI) was calculated as weight (kg) divided by height (m2). All measurements were performed in 2001. General and abdominal obesity were defined as BMI ≥ 30 kg/m^2^^18^ and WC ≥ 88 cm for women and ≥ 102 cm for men^19^, respectively.

### Diabetes, prediabetes and normal glucose tolerance definitions

Diabetes was defined as fasting blood sugar (FBS) ≥ 126 mg/dL or 2-h post prandial glucose (2hpp) ≥ 200 mg/dL or by using anti-diabetic agent’s consumption. Participants were classified to prediabetes if their FBS and 2hpp levels were between 100 and 125.9 mg/dL and 140 and 200 mg/dL, respectively. Normal glucose tolerance (NGT) was defined as FBS < 100 mg/dL and 2-hpp < 140 mg/dL^20^. Glucose tolerance status was defined either by the combination of FBS and 2hpp or by FBS only.

### Events ascertainment

Every 2 years, all participants were contacted by interviewers by phone call and asked about new CVD events. Participants were contacted by phone up to four time, and if there was no response, participants were visited at their homes by the interviewers. In each interview, first, participants’ identity was confirmed and then they were asked to answer the following queries: (1) being alive, (2) having been hospitalized for any reason and particularly cardiovascular events, and (3) experiencing any of the following neurological symptoms: hemiparesis, dysarthria, facial asymmetry, imbalance and transient monocular blindness. When the responses to at least one of the items was positive, any documents related to the event, physician diagnosis and the hospital’s name were obtained and the related questionnaire was completed. The same approach was applied for face-to-face interviews in 2007 and 2013. For participants who experienced any incident CVD event, the date of its onset was considered the end of follow-up and after that they were followed for death. Cardiovascular events were confirmed through checking the reported events with the registry database of the Surveillance Department, Isfahan Cardiovascular Research Center^17^.

Six outcome variables were considered in this study: All-cause mortality, CVD mortality, and incidence of CVD events, ischemic heart disease (IHD), myocardial infarction (MI) and stroke. IHD included the occurrence of fatal and non-fatal MI, sudden cardiac death (SCD) and unstable angina. MI was defined as the combination of fatal and non-fatal MI, stroke was defined as fatal and non-fatal stroke. CVD events were defined as fatal and non-fatal MI, fatal and non-fatal stroke, sudden cardiac death and unstable angina, based on modified criteria of WHO Expert Committee^21^. MI was considered present when at least two of the following criteria were met: (1) typical chest pain lasting more than 30 min, (2) elevation of ST (an isoelectric segment on the ECG which represents the interval between repolarization and depolarization of ventricular) > 0.1 mV in at least 2 adjacent electrocardiograph leads, and (3) an increase in serum level of cardiac biomarkers. We defined SCD as death within 1 h of onset of a witnessed cardiac arrest, or abrupt collapse not preceded by > 1 h of symptoms. Using the WHO criteria, we considered stroke as a rapid-onset focal neurological disorder persisting for at least 24 h that had a probable vascular origin. The diagnoses of any CVD events were confirmed by a special panel including four expert cardiologists and expert neurologist^17^.

### Statistical analysis

Out of 6323 participants in the ICS, 5432 subjects who had at least one follow-up were entered in the survival analysis. Baseline characteristics were expressed as mean ± standard deviation (SD) for continuous variables and number (percent) for categorical variables. Cox proportional hazards models were used to assess the association between diabesity phenotype and each outcome. All associations were examined in both crude and adjusted models. In the adjusted model, the confounding effects of age, SBP, TG, sex, smoking status, history of heart disease, history of high blood pressure and history of diabetes were controlled. These variables were selected from 20 possible confounding variables via the forward stepwise method. The explanatory variables are from the baseline at 2001 and the incidence of each event until 2017 was considered outcome variable. We also performed stratified analysis based on sex to remove its residual confounding effects on the associations. All of the analyses were performed by R software^22^, the cox model was run using the survival package^23^ with the Efron method for handling tied event times.

### Ethics approval and consent to participate

Participants were recruited from 2001 and followed-up for at least ten years. All subjects signed the informed consent form for the experimental procedure. Ethics approval was obtained from the Isfahan Cardiovascular Research Center Ethics Committee, a WHO collaborating center in the Eastern Mediterranean Region, and Isfahan University of Medical Sciences and conformed to the Declaration of Helsinki.

## Results

Table 1 shows the general characteristics of the participants based on diabesity phenotype defined by FBS and BMI. In all groups based on diabetes status, there were more females than males, but the educational level was lower in obese individuals compared with those with normal weight or overweight.

**Table 1:** Basic characteristic of participants by disability levels^1^.

Variable	Normal glucose		Pre-diabetes		Diabetes	
	Normal weight/overweight	Obese	Normal weight/overweight	Obese	Normal weight/overweight	Obese
Number (%)	3415 (63.14%)	836 (15.46%)	428 (7.91%)	200 (3.70%)	356 (6.58%)	174 (3.22%)
Female, n (%)	1490 (43.63%)	607 (72.61%)	228 (53.27%)	144 (72.00%)	188 (52.81%)	117 (67.24%)
Age (years)	49.92 ± 11.64	48.66 ± 10.01	54.39 ± 12.79	50.72 ± 10.76	56.20 ± 11.35	54.54 ± 10.80
Education, n (%)						
≤ 5 y	2344 (68.70%)	595 (71.34%)	331 (77.34%)	148 (74.37%)	277 (78.25%)	149 (85.63%)
6–12 y	821 (24.06%)	199 (23.86%)	77 (17.99%)	43 (21.61%)	65 (18.36%)	20 (11.49%)
> 12 y	247 (7.24%)	40 (4.80%)	20 (4.67%)	8 (4.02%)	12 (3.39%)	5 (2.87%)
Married, n (%)	3177 (93.03%)	739 (88.40%)	368 (85.98%)	177 (88.50%)	317 (89.04%)	151 (86.78%)
Current smokers, n (%)	666 (19.53%)	81 (9.70%)	48 (11.21%)	21 (10.50%)	46 (12.92%)	14 (8.09%)
Total physical activity (MET-min/week)	935.40 ± 566.07	775.51 ± 479.75	813.59 ± 549.03	741.37 ± 494.45	732.24 ± 503.93	703.93 ± 463.17
SBP	118.57 ± 19.90	123.74 ± 19.84	125.24 ± 22.63	130.47 ± 22.80	129.49 ± 21.71	136.55 ± 22.28
DBP	76.86 ± 10.97	80.29 ± 11.69	78.97 ± 11.02	83.31 ± 11.92	81.72 ± 12.09	85.37 ± 13.51
HDL	46.65 ± 10.20	47.06 ± 10.14	47.53 ± 10.72	47.12 ± 11.23	47.51 ± 11.43	48.30 ± 10.08
LDL	125.17 ± 42.83	133.23 ± 42.18	137.20 ± 43.83	132.69 ± 42.13	138.46 ± 45.09	139.69 ± 47.21
TG	173.85 ± 92.49	210.13 ± 102.90	202.86 ± 108.37	226.46 ± 104.17	241.68 ± 125.06	271.04 ± 139.05
Total cholesterol	206.56 ± 50.09	222.32 ± 50.73	225.31 ± 53.55	225.10 ± 49.37	234.30 ± 56.46	242.20 ± 58.07
Heart disease history, n (%)	205 (6.00%)	62 (7.42%)	30 (7.01%)	21 (10.50%)	43 (12.08%)	17 (9.77%)
High blood pressure history, n (%)	361 (10.57%)	147 (17.58%)	82 (19.16%)	56 (28.00%)	85 (23.88%)	60 (34.48%)
Diabetes history, n (%)	20 (0.59%)	16 (1.91%)	12 (2.80%)	17 (8.50%)	231 (65.07%)	95 (54.60%)
FBS (mg/dL)	77.91 ± 9.01	79.69 ± 9.05	96.09 ± 13.90	98.88 ± 12.91	166.96 ± 63.94	152.18 ± 54.41
2hpp (mg/dL)	91.11 ± 17.76	95.75 ± 18.59	145.24 ± 28.49	147.92 ± 26.64	265.55 ± 88.66	257.05 ± 77.00
BMI (kg/m^2^)	24.77 ± 3.09	33.84 ± 6.24	25.43 ± 3.08	33.69 ± 4.59	25.96 ± 2.68	34.44 ± 7.75
WC (cm)	90.60 ± 10.77	106.21 ± 11.70	93.06 ± 12.51	106.86 ± 9.83	96.46 ± 9.71	109.83 ± 10.97
HTW	660 (19.33%)	514 (61.56%)	145 (33.88%)	130 (65.00%)	153 (42.98%)	134 (77.01%)

*SBP* systolic blood pressure, *DBP* diastolic blood pressure, *HDL* high-density lipoprotein, *LDL* low-density lipoprotein, *TG* Triglycerides, *BMI* body mass index, *WC* waist circumference, *FBS* fasting blood sugar, *2hpp* 2-h post prandial glucose, *HTW* hyper-triglyceridemic waist.

Obesity was defined by body mass index.

^1^Values are mean ± SD unless indicated.

Subjects with normal weight and normal glucose level had the highest physical activity level while patients with diabetes and obesity had the lowest level (935.40 + 566.07 vs. 703.93 + 463.17 MET.min/wk). Independent of diabetes status, obese subjects had greater SBP, DBP, and TG levels compared with those with normal weight or overweight. Diabetic and pre-diabetic participants were older than participants with NGT. In all three categories based on glucose tolerance status, the proportion of females, and participants with lower educational level and lower physical activity level were higher in obese individuals compared to non-obese participants.

After a median follow-up of 11.25 years, a total of 819 CVD events (including 647 IHD, 165 MI, and 172 stroke), 181 CVD deaths and 488 all-cause deaths were recorded.

Table 2 shows crude and adjusted HRs (95% CIs) for CVD events for each variable. All variables were independently and significantly associated with the risk of CVD in the crude model except for the phenotypes of obese subjects either with NGT or prediabetes, current smoking, and HDL. However, adjustment for potential confounders weakened the association but just disappeared significance only for the phenotype of prediabetes-non-obese. Also, we tested the interaction term between diabetes and obesity and it was not significant.

**Table 2:** Crude and adjusted HRs (95% CIs) for CVD based on each variable^1^.

Variable	Crude model		Adjusted model	
	HR (95% CI)	p-value	HR (95% CI)	p-value
Diabesity^1^				
NGT & nonobese	Ref	–	Ref	–
NGT & obese	0.90 (0.72, 1.11)	0.30	0.90 (0.72, 1.12)	0.35
Prediabetes and nonobese	1.40 (1.09, 1.81)	0.01	0.95 (0.73, 1.23)	0.68
Prediabetes and obese	1.18 (0.82, 1.69)	0.36	0.96 (0.66, 1.39)	0.82
Diabetes and nonobese	2.86 (2.33, 3.51)	< 0.01	1.55 (1.17, 2.05)	< 0.01
Diabetes and obese	2.63 (1.99, 3.49)	< 0.01	1.42 (1.01, 1.99)	0.04
Female	0.79 (0.69, 0.91)	< 0.01	0.76 (0.64, 0.89)	< 0.01
Age (years)	1.06 (1.05, 1.07)	< 0.01	1.05 (1.04, 1.05)	< 0.01
Education				
≤ 5 year	Ref	–		
6–12 year	0.62 (0.52, 0.74)	< 0.01		
> 12 year	0.59 (0.43, 0.81)	< 0.01		
Married	0.60 (0.48, 0.74)	< 0.01		
Current smokers	1.16 (0.98, 1.39)	0.09	1.46 (1.19, 1.80)	< 0.01
Total physical activity	1.00 (1.00, 1.00)	< 0.01		
SBP	1.02 (1.02, 1.03)	< 0.01	1.01 (1.01, 1.02)	< 0.01
DBP	1.03 (1.03, 1.04)	< 0.01		
HDL	1.00 (0.99, 1.01)	0.78		
LDL	1.00 (1.00, 1.01)	< 0.01		
TG	1.00 (1.00, 1.00)	< 0.01	1.00 (1.00, 1.00)	0.04
Total cholesterol	1.00 (1.00, 1.01)	< 0.01		
Heart disease history	2.91 (2.43, 3.50)	< 0.01	2.28 (1.86, 2.80)	< 0.01
High blood pressure history	3.03 (2.61, 3.52)	< 0.01	1.30 (1.06, 1.60)	0.01
Diabetes history	2.86 (2.38, 3.45)	< 0.01	1.56 (1.09, 2.22)	0.01
Diabetes				
NGT	Ref	–		
Prediabetes	1.36 (1.10, 1.68)	< 0.01		
Diabetes	2.85 (2.40, 3.38)	< 0.01		
Obesity defined by BMI	0.98 (0.83, 1.15)	0.78		
Obesity defined by WC	1.08 (0.94, 1.24)	0.27		
HTW	1.39 (1.21, 1.60)	< 0.01		

^1^Obesity was defined by body mass index.

**Figure 1:**
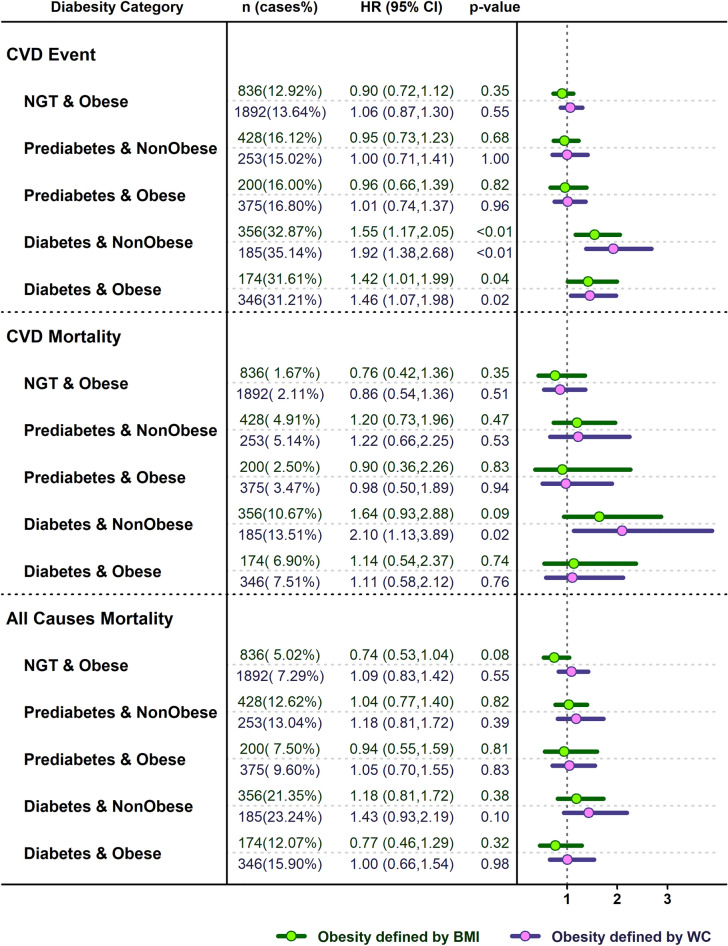
HRs (95% CIs) of adjusted model for CVD events, CVD mortality and all-cause mortality according to the diabesity phenotypes. Values are adjusted for age, SBP, TG, sex, smoking status, history of heart disease, history of high blood pressure and history of diabetes.

Figure 1 shows the adjusted HRs and 95% confidence intervals (CIs) for incident CVD events, CVD mortality and all-cause mortality across different diabesity phenotypes. Irrespective of obesity status (yes or no) and phenotype (general or abdominal), patients with diabetes had greater risk for incident CVD events in comparison with NGT-non obese subjects.

**Figure 2:**
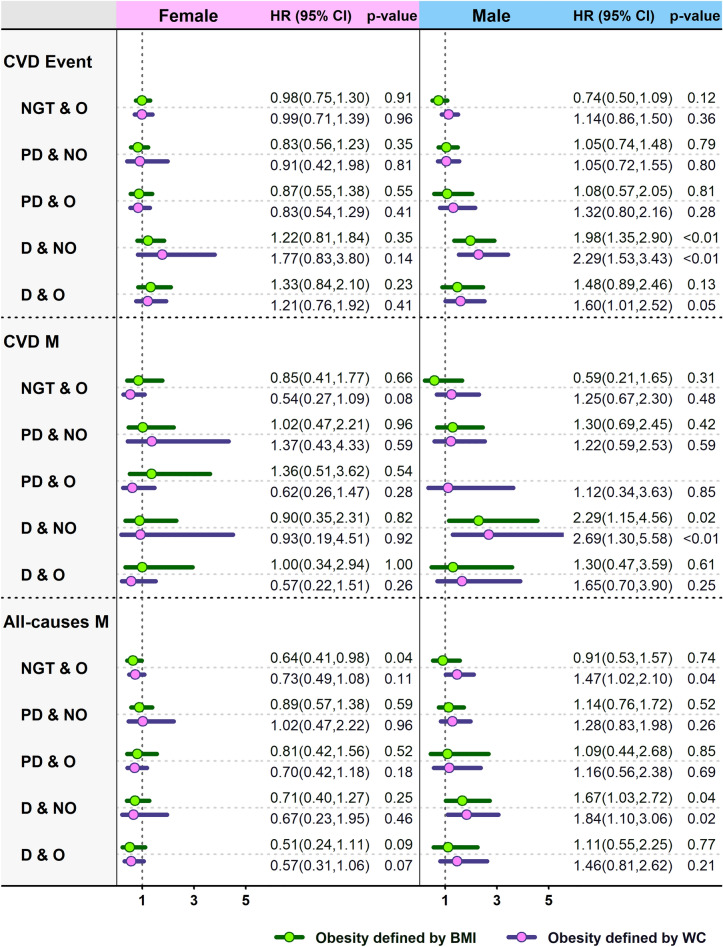
Sex-stratified HRs (95% CIs) of adjusted model for cardiovascular events, cardiovascular mortality and all-cause mortality according to the diabesity phenotypes. Values are adjusted for age, SBP, TG, smoking status, history of heart disease, history of high blood pressure and history of diabetes.

Figure 2 illustrates the association between diabesity phenotypes and the risk of incident CVD events, and CVD and all-cause mortality stratified by sex. In males, diabetes doubled CVD incidence risk in non-obese subjects (either based on general or abdominal adiposity), whereas in obese individuals, it tended to increase incident CVD events risk by 60% only in those with abdominal obesity in comparison with those with non-obese, normal glucose tolerance test. In females, diabetes increased the risk of CVD mortality by more than twofold only in subjects who were not abdominally or generally obese, while in obese subjects, no significant association was observed.

**Figure 3:**
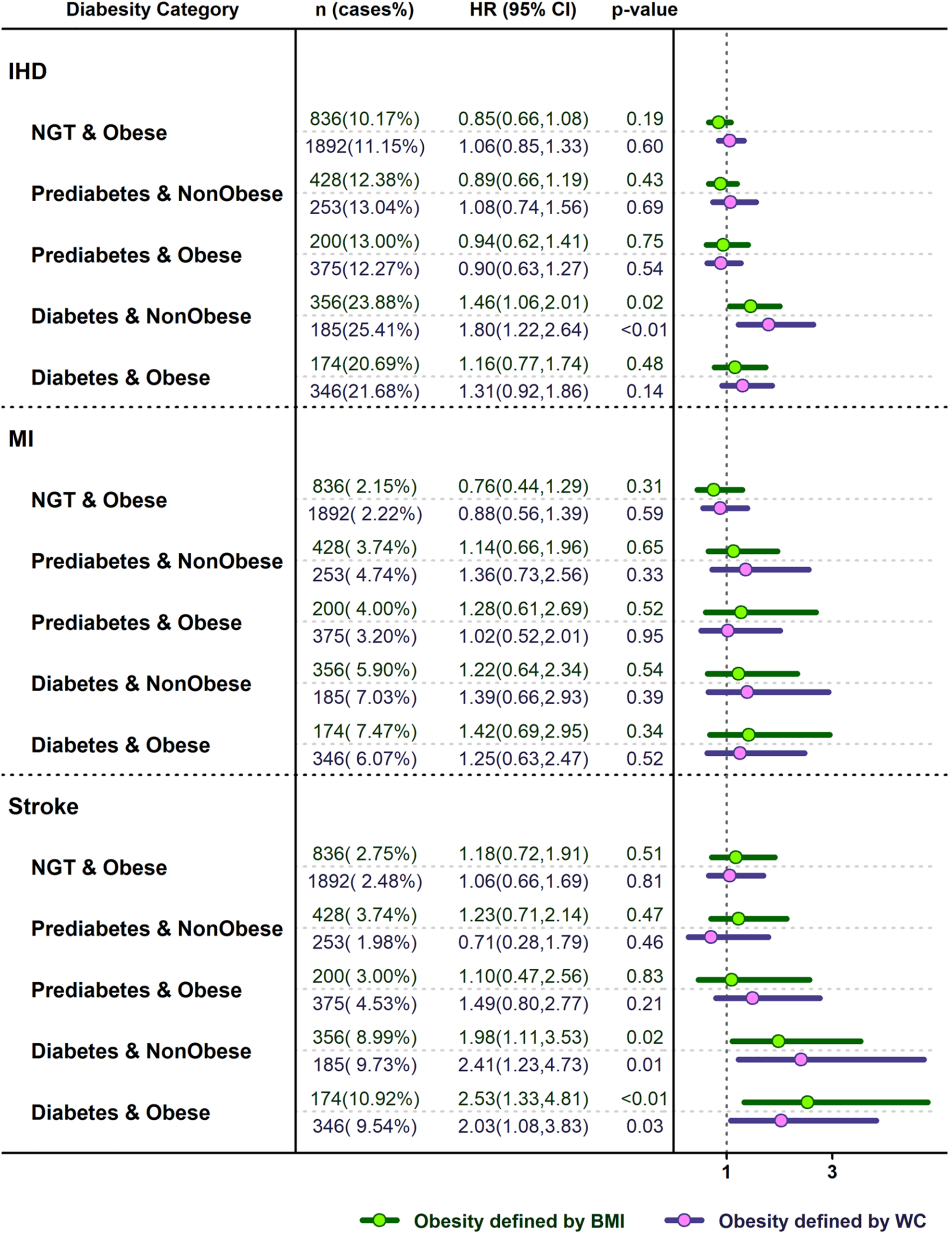
HRs (95% CIs) of adjusted model for various cardiovascular events according to the diabesity phenotypes. Values are adjusted for age, SBP, TG, sex, smoking status, history of heart disease, history of high blood pressure and history of diabetes.

**Figure 4:**
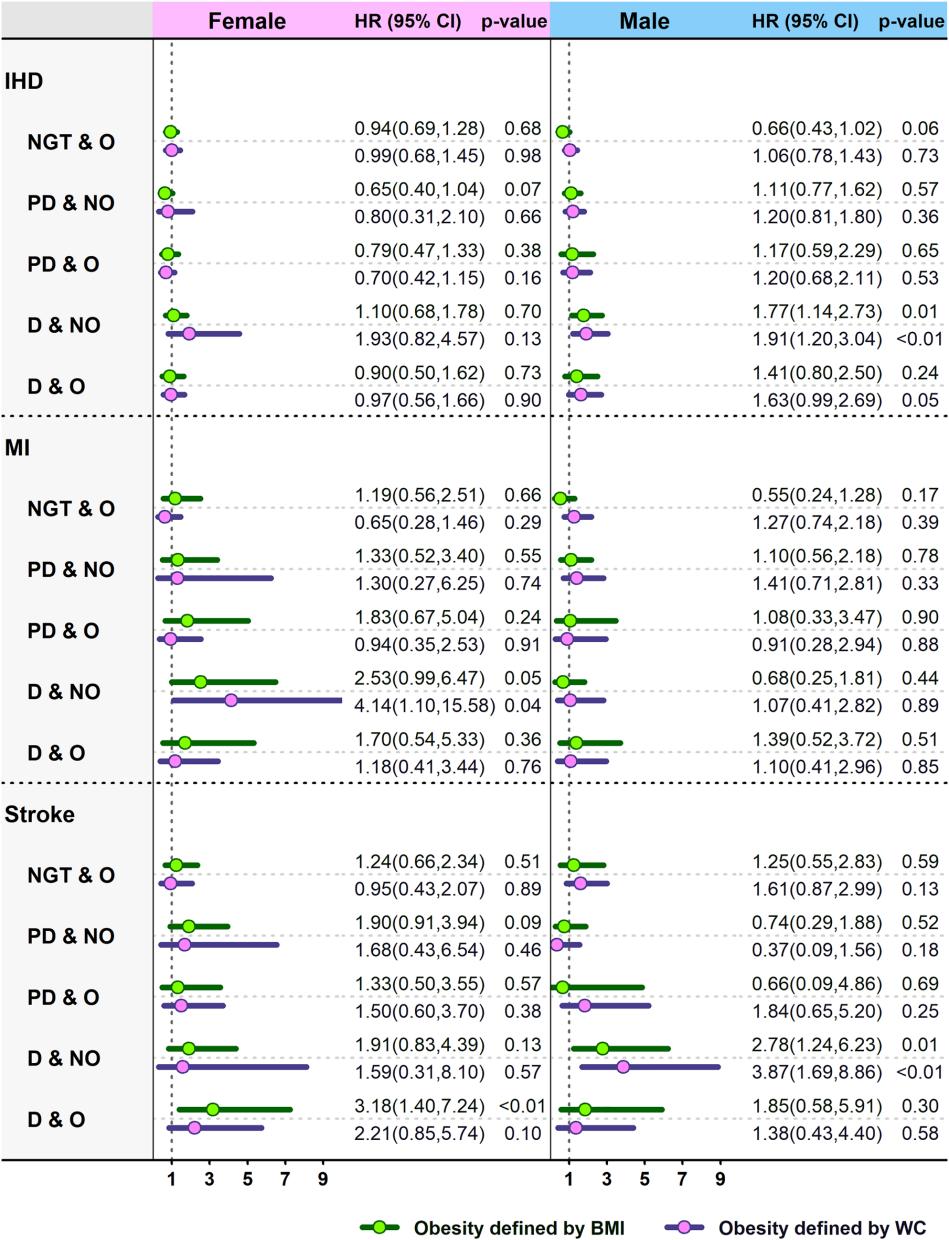
Sex-stratified HRs (95% CIs) of adjusted model for various cardiovascular events according to the diabesity phenotypes. Values are adjusted for age, SBP, TG, sex, smoking status, history of heart disease, history of high blood pressure and history of diabetes.

Figure 3 presents adjusted HRs and 95% CIs for the incidence of various CVD events across different diabesity phenotypes. Independent of obesity definition, having diabetes was associated with increased risk of incident stroke in both non-obese and obese subjects. Regarding IHD, diabetes was associated with increased risk of IHD only in non-obese subjects in comparison with those with NGT, non-obese. No significant association was found for MI. Figure 4 shows the same association stratified by sex.

## Discussion

In this prospective study of Iranian adults, we found that the phenotypes of diabetes-obese and diabetes-non obese were associated with an increase in the risk of incident CVD events, CVD mortality and all-cause mortality. In contrast, the phenotypes of NGT-obese, prediabetes-obese and pre-diabetes-non obese were not associated with the incident risk of the same outcomes in comparison with NGT-non obese phenotype. The findings for diabetes-obese and diabetes-non obese were independent of obesity definition. In analyses stratified by sex, only in males with abdominal obesity and DM, diabesity was associated with increased risk of CVD events.

To our knowledge, there is only one previous study exploring the association of diabesity and CVD^16^. In line with our findings, this study showed that diabesity, defined either by general or by abdominal obesity, increased the risk for incident CVD, while no association was observed for prediabetes^16^. However, in contrast with their findings, we found that general and abdominal obesity were not a risk factor for any of the outcomes when serum glucose levels were normal. Our study differs from the earlier one in some ways. While our study population were more likely to be obese, prediabetes and DM were more prevalent in Chinese. The corresponding values for obesity were around 50% in Iranians and almost 10% in Chinese. These differences in studies population suggest the relevance of performing such studies in different populations. Furthermore, follow-up duration in our study was considerably longer than the earlier one (13 vs. 4.5 year). Since the risk of age-adjusted macrovascular events rose by around 50% for each 5-year increment in the duration of T2DM^24^, therefore, our study with longer duration of follow-up can provide greater statistical power to detect an association.

Although no previous study has examined the association of diabesity with CVD and death among Iranians, an earlier study examined the association of different anthropometric measures with mortality in patients with diabetes^25^. They suggested that assessing BMI alone has some limitations for predicting the risk of CVD and mortality incidence and it would be more precise when considered alongside other measurements of obesity, that is, WC and hip circumference. Bozorgmanesh et al. demonstrated that in higher values of BMI, the waist to hip ratio decreased, which is resulted from an increase in hip circumference^25^. Greater hip circumference is a protective factor for CVD events per se, whereas enlarged WC reflects abdominal or intraabdominal fat accumulation which are associated with dyslipidemia and insulin resistance^26^. In line with this study, we observed that the association between DM and CVD was stronger in non-obese subjects in comparison with obese ones.

While our results showed no association between diabesity and CVD mortality, we observed that subjects with DM but not abdominal obesity had increased risk for CVD mortality. This might be attributable to enlarged hip circumference in obese subjects which possibly plays a protective role, whereas non-obese subjects are deprived from its beneficial effects. Another explanation for the null association between diabesity and death from CVD and all-cause mortality might be owing to hypertriglyceridemic waist (HTW) phenotype^27^. In the Tehran Lipid and Glucose Study (TLGS), the HTW phenotype was associated with increased risk of CVD, but not CVD and all-cause mortality^27^. In the present study, we failed to control the effect of HTW due to its high collinearity with TG, however, it is suggested that further studies examine its association with various outcomes in diabetic patients in particular.

Based on subgroup analysis, the association of diabesity with the outcomes assessed appeared to be sex-specific. Sex-stratified results indicated that the presence of DM was associated with increased risk of CVD events in males with abdominal, but not general, obesity. Nevertheless, in women, diabesity was not associated with the risk of any outcomes. This might be explained, at least to some extent, by variations in fat distribution in males and females, which is determined by higher fat accumulation in gluteofemoral region in female and more truncal fat in males^28^. Moreover, it has been well-established that differences in factors related to gender, such as cultural, behavioral, mental and socioeconomic status account, to various degrees, for variations between men and women for CVD outcomes^29^.

The mechanisms through which diabesity increases the risk of CVD events are likely to overlap with those which have been established for diabetes and obesity^30^. Excess visceral adiposity can cause ectopic fat accumulation in heart, liver, or blood vessels, leading to lipotoxicity, prothrombotic status, non-calcified plaques formation and chronic inflammation^30^. Elevated inflammatory markers stimulate LDL-c oxidation per se, which promotes atherosclerosis process^30^. Moreover, diabetes and age-induced senescence are associated with endothelial dysfunction caused by reactive oxygen species (ROS), inflammatory mediators and inducible nitric oxide. This, in turn, produces advanced glycation end products (AGEs) which stimulate mitogen-activated protein kinase (MAPK) and NFκB cascades, leading to elevated production of ROS, inflammatory, profibrotic and prothrombotic factors^30^. These factors cause arterial stiffness, vascular calcification, and induce plaque accumulation in vessels^30^.

Our results showed that diabetes increased the risk of CVD events and CVD mortality in subjects who were not obese, whereas the diabesity phenotype was not associated with the risk of CVD mortality. The null association between diabesity and CVD and all-cause mortality might be explained by “obesity paradox”. There is a large body of evidence indicating lower risk of mortality in obese patients with established CVD in comparison with lean counterparts. Indeed, it seems that the relationship between obesity and CVD death is influenced by cardiac fitness rather than fat mass reduction alone^31^. In support of this, in a large prospective study on men with coronary heart disease, the relationship between obesity and mortality greatly depended on cardiovascular fitness, suggesting that assessing adiposity regardless of fitness cannot be an appropriate predictor of mortality risk in patients with CVD^32^.

The limitations of this study include lack of repeated measures of adiposity and not considering the influence of weight change on the outcomes over the follow-up duration. In addition, despite adjustment for various risk factors, we cannot rule out the possibility of residual and unmeasured confounders. Another point to consider is that subjects with general obesity usually have enlarged waist circumference and vice versa. Therefore, it was not possible to examine the independent influence of each of these measures and it should be considered when interpreting the results. Furthermore, examining a combination of various anthropometric measures would be more appropriate rather than only one. However, since the primary aim of our study was evaluating the associations for diabesity which is defined based on BMI, we just assessed one indicator for defining obesity status. The lack of data on the duration of having diabetes and glycemic control in the study population were other limitations of our study. Our study has its own strengths including the prospective study design with a long-term duration of follow-up, recruiting participants with a wide age range, exploring associations based on participants’ sex, examining relationships based on CVD events, and defining diabesity based on both abdominal and general obesity.

## Conclusion

Our results show that although diabetes, irrespective of obesity definition and presence, is a predictor of CVD events, its predictive value for CVD mortality varies by obesity status. DM was associated with increased risk of CVD mortality only in subjects who were not abdominally obese. These associations differed by sex. DM was associated with increased risk of incident CVD events, CVD mortality and all-cause mortality only in males who were not generally or abdominally obese. In contrast, no association was found in obese males or obese and non-obese females. Further studies are needed to explore the association between diabesity and CVD and mortality and its mediating factors.

## Data Availability

The datasets used in the current study are available from the corresponding author upon reasonable request. A representative deidentified of it is available from the figshare database (https://figshare.com/articles/dataset/CVD_risk_assessment_sample_dataset/5480224).

## Abbreviations

2hpp: 2-Hour post prandial glucose
AGEs: Advanced glycation end products
BMI: Body mass index
CI: Confidence interval
CVD: Cardiovascular disease
DM: Diabetes mellitus
FBS: Fasting blood sugar
HR: Hazard ratio
ICRC: Isfahan cardiovascular research center
ICS: Isfahan cohort study
IDF: International diabetes federation
IHD: Ischemic heart disease
MAPK: Mitogen-activated protein kinase
MI: Myocardial infarction
NGT: Normal glucose tolerance
ROS: Reactive oxygen species
SCD: Sudden cardiac death
SD: Standard deviation
WC: Waist circumference
WHO: World health organization
